# Mitigating Ethnic Moral Disengagement: The Role of Inhibitory Control, Cognitive Reflection, and Growth-Oriented Personal Values from an Integrative Perspective

**DOI:** 10.3390/bs15020169

**Published:** 2025-02-04

**Authors:** Giuseppe Corbelli, Marinella Paciello, Carmela Sportelli, Paolo Giovanni Cicirelli, Francesca D’Errico

**Affiliations:** 1 Department of Psychology, Sapienza University of Rome, 00185 Rome, Italy; giuseppe.corbelli@uniroma1.it; 2 Faculty of Psychology, Uninettuno Telematic International University, 00186 Rome, Italy; carmela.sportelli@uninettunouniversity.net; 3 For.Psi.Com Department, Università degli Studi di Bari “Aldo Moro”, 70121 Bari, Italy; paolo.cicirelli@uniba.it (P.G.C.); francesca.derrico@uniba.it (F.D.)

**Keywords:** cognitive reflection, growth mindset, inhibitory control, moral disengagement, personal values

## Abstract

Despite the consequences of ethnic moral disengagement, such as ethnic bullying, racism, and prejudice, a comprehensive understanding of how to effectively counter it remains an ongoing area of research. The present study proposes an association between ethnic moral disengagement and three individual dimensions: the executive function of inhibitory control, a reflective cognitive style, and personal values that reflect growth-oriented motivations in contrast to self-defensive ones. By evaluating these dimensions respectively through a behavioral task, a cognitive measure, and a self-report instrument, the aim is to understand the role of basic behavioral capability, cognitive reflection, and growth-oriented values in reducing ethnic moral disengagement. The study, conducted on 413 participants (243 female, M = 19.60 years, SD = 1.46) using a structural equation modeling approach, found that while inhibitory control was not significantly linked to ethnic moral disengagement, reflective information processing and broader value horizons may constitute a key resource for opposing it. Overall, these results suggest that individuals who adopt such a reflective and growth-oriented mindset may elaborate differences and unfamiliar encounters as opportunities rather than as threats to be defended against by justifying themselves and externally displacing responsibility for their decisions and actions.

## 1. Introduction

Moral disengagement has been defined as the set of social cognitive mechanisms used to restructure the meaning of aggressive and immoral behavior and its effects and the sense of personal accountability in order to bypass moral control (Bandura, 1991). In accordance with social cognitive theory, the investigation of moral disengagement (MD) in the context of ethnic prejudice has proven essential for understanding how mechanisms related to the ethnic background of potential victims are associated with racist and aggressive behaviors (Iannello et al., 2021; Lo Cricchio et al., 2021; Papotti & Caravita, 2024). This line of research is critical for identifying strategies to better understand the perpetration of intolerance and discriminatory behaviors. Indeed, studies that have investigated the determinants of aggressive behavior directed toward migrants have demonstrated the key role of cognitive distortions and prejudicial ways of thinking (Bayram Özdemir et al., 2020; D’Errico & Paciello, 2018), showing how a greater presence of such cognitions increases the likelihood of harassment directed toward them. Moreover, research has demonstrated that children and adolescents exhibiting high levels of MD tend to experience negative sentiments toward their peers from different ethnic backgrounds (Iannello et al., 2021). This phenomenon has also been observed in the context of more negative attitudes towards immigrants (Bayram Özdemir et al., 2024). In the context of bullying, research has indicated differences in self-justifying bullying depending on immigration status (Caravita et al., 2019) and that ethnic MD is associated with ethnic bullying and ethnic cyberbullying (Lo Cricchio et al., 2021). Prejudices and stereotypes may activate specific MD mechanisms, such as the dehumanization of victims, which compels bullies to act more cruelly and harshly toward ethnically diverse individuals (Bandura, 2016; Webster & Saucier, 2015).

Therefore, despite the detrimental consequences of ethnic MD, such as ethnic bullying and racism, a thorough understanding of the individual dimensions linked with the activation of ethnic moral disengagement remains an ongoing area of research. Although existing research addresses individual antecedents of moral disengagement (Detert et al., 2008; Moore, 2015; Ogunfowora et al., 2022), an integrated approach that considers individual dimensions of different types together is currently lacking for both general and specific ethnic contexts. In the present work, the aim is to contribute to the emerging literature on this specific type of moral disengagement in terms of antecedents (i.e., ethnic moral disengagement) by adopting a perspective that integrates three different levels of analysis: (1) basic personality dimensions related to behavioral regulation, such as inhibitory control; (2) cognitive dimensions, such as the propensity forreflective thinking; and (3) motivational dimensions, such as the person’s value structure. These three levels could help us understand whether ethnic moral disengagement is more influenced by dispositional difficulties in behavioral regulation, different styles of cognitive processing, or culturally learned individually salient motivations, such as values. This contemporary examination of behavioral, cognitive, and motivational dimensions is necessary because studies often focus on a specific aspect of individual functioning without considering them simultaneously. In fact, these dimensions operate within the personality as an integrated system and are organized coherently (Caprara & Cervone, 2000). As an integrated system of functioning in which motivational dimensions, modes of thinking, and behavioral tendencies operate together, the specific contribution of each dimension must be examined in light of their mutual influences.

Thus, to understand the role these variables play toward ethnic moral disengagement, considering their presence and relationship together could provide insights into which aspects are most effective for intervening to mitigate such ethnically-based biases. For example, the present study aims to explore whether it is a priority to reinforce a reflective cognitive style or to promote inclusive and prosocial personal values that foster moral attitudes toward individuals from groups different from one’s own. Such an approach can be particularly useful in understanding the possible role of personal dimensions related to basic self-regulatory processes or socially learned motivational orientations in contributing to ethnic moral disengagement. The overall hypothesis, therefore, is that a personality system capable of inhibiting impulsive responses, reflecting carefully on reality, and projecting toward a values-based mindset of personal and collective growth can reduce the reliance on ethnic moral disengagement that allow individuals to engage in discriminatory and racist attitudes and actions.

### 1.1. Inhibitory Control and Ethnic Moral Disengagement

Inhibitory control, an executive function rooted in temperament, refers to the ability to suppress dominant or maladaptive responses (Rothbart et al., 2001). This capacity is essential for self-regulation and has a significant influence on behavior across various domains. In the case of moral regulation, the capacity to control impulses and restrain behavioral responses in the face of temptations and pressing situations is crucial for thoroughly considering the pros and cons of one’s actions and regulating behavior in a way that is goal-oriented and focused on long-term benefits for both the individual and the collective. It has been observed that individuals with poor self-control are at greater risk of engaging in aggressive and antisocial behaviors (Gottfredson & Hirschi, 1990; Nedelec & Beaver, 2014), while those capable of exercising inhibitory control are more likely to engage in prosocial and reparative behaviors (Colasante et al., 2014; Luengo Kanacri et al., 2013).

Studies examining the relationship between inhibitory control and moral disengagement remain limited. However, the importance of the ability to inhibit impulsive responses in connection with ethical and moral behaviors is acknowledged, in line with Bandura’s theory of moral agency (Bandura, 1990, 1991), emphasizing that the capacity to inhibit inappropriate behaviors represents one of the pathways of moral regulation. Thus, it is reasonable to think that in the same way that high levels of inhibitory control promote moral regulation, low levels of self-control might incentivize moral disengagement. One recent and interesting longitudinal study by Gulseven and colleagues (Gülseven et al., 2023) highlighted the negative developmental relationship between moral disengagement and self-control, suggesting that youth with strong self-control skills are better equipped with the necessary tools to resist social pressures that lead to moral disengagement. Similarly, studies focusing on basic personality dimensions associated with impulsivity (Paciello et al., 2023), irritability (Caprara et al., 2014), and trait-anger (Rubio-Garay et al., 2016) indicate that difficulties in managing impulses can foster moral disengagement. Moreover, the ability to control impulsive responses is also associated with beliefs in self-efficacy regarding behavioral regulation (Favini et al., 2024). This relationship between ability and perceived ability within the context of inhibitory control is particularly interesting, as beliefs in self-efficacy represent one of the individual protective dimensions against moral disengagement most studied within the social-cognitive approach (Bandura, 2001). Examples include beliefs in the ability to resist deviant pressures (Fida et al., 2018) and to exercise moral behavioral control (Alexandra, 2019). Moreover, within the context of ethnic prejudice, disengagement may be supported by strongly activating hostile emotions (D’Errico & Paciello, 2018). In these cases, inhibitory control is important to avoid the activation of mechanisms facilitated by negative and activating feelings, which drive discriminatory attitudes and behaviors that lead to impulsive aggressive responses. Overall, these studies suggest a possible link between the lack of impulse control and ethnic moral disengagement, probably tied to reactive and impulsive harmful responses against immigrants, which are justified through moral disengagement. Therefore, the following can be hypothesized:

**H1.**  *Higher inhibitory control is associated with lower ethnic moral disengagement.*

However, it is important to consider that, in light of current knowledge, studies in this area have often relied on self-reported assessments rather than objective tests of inhibitory processes. Therefore, it would be essential to examine the role of such basic executive functions using objective tools to better understand how to hinder moral disengagement. Furthermore, it is not certain that the relationship is linear, as some researchers emphasize the importance of examining this connection by considering other individual dimensions (e.g., metacognitive abilities and worldviews) within a broader view of the moral regulation system. In fact, while certain dimensions might facilitate the translation from impulse to action through moral disengagement, others might hinder it. An example is the tendency toward hostile and vengeful cognitions (Caprara et al., 2014) or negative worldviews, such as social cynicism (Alexandra, 2019), which facilitate the use of moral disengagement mechanisms and harmful behaviors in the presence of difficulties in impulse control. In contrast, dimensions related to awareness and self-reflection can inhibit reliance on moral disengagement and impulsive responses (Georgiou et al., 2020).

Finally, it is important to consider the type of moral disengagement under study, namely ethnic moral disengagement. There are no studies that have directly investigated this specific moral disengagement (related to racism, aggression, and ethnic victimization) and its connection to self-control dimensions. Thus, exploring this particular relationship could help clarify whether mechanisms of ethnic moral disengagement are mainly hot cognitive processes triggered by internal impulses that individuals cannot control or whether they represent more cold processes linked to racist instrumental behaviors and/or attitudes. Recent studies seem to support the hypothesis that ethnic victimization and harassment are associated with higher levels of impulsivity, especially when individuals also hold anti-immigrant views (Bayram Özdemir et al., 2018). Furthermore, Bayram Özdemir and colleagues (Bayram Özdemir et al., 2020) suggest that problems with impulse control play a lesser role in engagement in ethnic victimization, as ethnic victimization appears to be a more deliberate behavior, and for this reason, attitudes and values, rather than basic self-regulatory skills, could be more influential.

### 1.2. Heuristic Reasoning and Ethnic Moral Disengagement

Heuristic processing, often classified under the framework of system 1 thinking, relies on automatic and relatively effortless judgment processes that can facilitate rapid evaluations without extensive reflective oversight (Kahneman, 2012). By contrast, reflective processing (or system 2 thinking) is characterized by deliberate, rule-governed reasoning that demands sustained cognitive engagement (Evans, 2008; Kahneman, 2012; Toplak et al., 2014). Also, in the moral field, a substantial body of empirical work indicates that this lower-effort processing fosters moral simplifications and cognitive shortcuts (Gigerenzer & Gaissmaier, 2011). A possible theoretical explanation involves the minimal introspection inherent in heuristic thinking, which rapidly legitimizes biased group perceptions or discriminatory norms, avoiding the more extensive and effortful cognitive moral evaluations associated with reflective reasoning (Pennycook et al., 2015). As a result, individuals can more smoothly adopt self-serving interpretations to reduce negative self-reactions and preserve a favorable self-image. Evidence from Pennycook and colleagues (Pennycook et al., 2015) supports the notion that individuals exhibiting higher levels of cognitive reflection are likely to produce complex and well-considered moral judgments. Bustamante and Chaux (Bustamante & Chaux, 2014) suggest how the development of thorough and effortful reasoning aimed at analytically evaluating different arguments before making moral judgments can counteract the use of cognitively biased processes such as moral disengagement. In addition, Zhao and colleagues (Zhao et al., 2021) recently corroborated the link between cognitive impulsivity and moral disengagement. This allows us to think that a reflective cognitive style offers a buffering effect against unexamined mental shortcuts and other distortions that heighten moral disengagement. This is in alignment with Bandura’s (Bandura, 1991; Bandura et al., 1996) observation that moral agency is preserved through cognitive and behavioral self-regulation but that these are all made possible by self-reflection of the complexities of the personal, situational, and behavioral determinants of behavior. Such self-vigilance acted upon one’s own morally charged thoughts and processes, by definition, requires a certain degree of effort, thus ascribing this type of cognitive processing to a more effortful and reflective type 2 system. Indeed, this reflective reasoning process, by its very nature, can potentially involve a high cognitive cost, as such self-monitoring can lead to openly acknowledging one’s cognitive distortions compared to one’s moral standards (Bandura et al., 1996).

Therefore, such effortful and attentive reasoning can be undermined when heuristic processes predominate and allow for rapid simplification of ethically questionable behavior, especially one based on a stark and morally charged distinction between ingroup and outgroup (Lo Cricchio et al., 2022). Heuristic reasoning may thus accelerate the adoption of cognitive distortions that facilitate prejudice and lower empathic reasoning, particularly in the case of group-based biases (D’Errico et al., 2023). Conversely, reliance on more slow, effortful, and reflective reasoning could prove itself useful to better shield the individual from the adoption of these readily accessible ethnic prejudices and biases (Gawronski & Payne, 2010; Houwer, 2014; Strack & Deutsch, 2004), ultimately mitigating the self-justifications that enable ethnic moral disengagement.

Hence, we propose that stronger reflective tendencies are linked with diminished ethnic moral disengagement, as a more reflective approach could interrupt biased justifications:

**H2.**  *A reflective cognitive style, i.e., a higher propensity to engage in reflective reasoning, is negatively associated with ethnic moral disengagement.*

When moral evaluation revolves around ethnic outgroups, these self-protective quick and low-effort cognitive simplifications could be particularly robust, deflecting more reflective moral thinking through biased interpretations of intergroup behavior. In line with this perspective, relevant studies have shown the negative relationship between ethnic prejudice and a more analytical, slow, controlled mode of thinking (Blanchar & Sparkman, 2020; Yilmaz et al., 2016), and still, other studies have revealed how individuals with a stronger predisposition toward reflexivity tend to show lower levels of intergroup prejudice and a higher inclination toward intergroup contact (Hurtado, 2005). Individuals relying more on heuristic processes may be especially prone to adopting such prejudicial strategies because they apply lower scrutiny to the moral implications and complexities of their judgments, thereby lessening their personal accountability against the harm done to a member of the outgroup. Conversely, individuals predisposed to reflective processing likely should be able to slow down and eventually be able to examine the consequences of their intergroup attitudes, recognizing and thus being able to undermine the cognitive distortions that fuel ethnic moral disengagement.

### 1.3. Growth-Oriented Personal Values and Ethnic Moral Disengagement

Personal values can be regarded as guiding principles that identify individuals’ motivational attitudes and direct their judgments of right and wrong, acting as a broad moral compass (Schwartz, 1992, 2012). According to Schwartz’s theory, ten basic universal values reflect shared human motivations; these ten values are arranged along two orthogonal axes, one ranging from openness to change to conservation and the other from self-transcendence to self-enhancement, creating a quasi-circumplex structure that reflects the dynamic relations and mutual compatibilities among them (Vecchione et al., 2009). Values that are closer tend to be more conceptually compatible, whereas those opposite each other are increasingly conflicting (Schwartz, 1992). This conceptualization has received broad empirical support, and the resulting value system is considered universally applicable, although the relative importance attributed to specific values may vary across individuals, groups, and cultural contexts (Caprara et al., 2012; Roccas & Sagiv, 2010; Schwartz, 2007). Extensive research has linked personal values to moral functioning, examining how relative differences in the importance attributed to values influence moral judgments, decisions, and actions (Caprara et al., 2012; Maio, 2010; Schwartz, 2007). Personal values also appear to play a significant role in the expression of covert and explicit forms of ethnic prejudice. Falanga and colleagues (Falanga et al., 2015) demonstrated that, in a sample of Italian adolescents, self-transcendence and openness to change were negatively associated with subtle and blatant prejudice toward Africans, whereas conservation and self-enhancement values were positively associated with these forms of prejudice. These results align with the findings of Davidov and colleagues (Davidov et al., 2008) on attitudes toward immigration: their study indicates that self-transcendence has a positive influence on support for immigration, while conservation has a negative effect. Wolf and colleagues (Wolf et al., 2019, 2021) further supported these findings, providing deeper insights into the role of perceived personal values of immigrants. Favorable attitudes toward immigrants tend to increase among individuals who strongly endorse self-transcendence values and de-emphasize self-enhancement values, particularly when they perceive immigrants as prioritizing self-transcendence over self-enhancement. Conversely, prejudice against immigrants intensifies among individuals with strong conservation values (e.g., security) who view immigrants as emphasizing openness to change (e.g., freedom), highlighting the impact of perceived value dissimilarity. Regarding studies on MD and values, the limited research in this area indicates that self-transcendence values are negatively associated with moral disengagement, whereas self-enhancement values are positively associated (Albouza et al., 2020; Paciello et al., 2013).

Various organizations of personal values into higher-order groupings, with different explanatory roles for understanding personal and social dynamics being theorized. One such organization is a one-dimensional, dipolar arrangement that groups the ten values according to their role in addressing anxiety arising from uncertainty. On one side of this dimension, a growth-oriented value mindset is found, encompassing values that promote openness to new experiences and foster curiosity and potential for personal growth. This mindset includes values such as hedonism, stimulation, self-direction, universalism, and benevolence. In contrast, at the other end of this bipolar dimension lies a self-protective value mindset, which is driven by anxiety and seeks stability, control, predictability, and security as a defense against uncertainty (Schwartz, 2012). Consequently, individuals for whom the value structure oriented toward expansion and growth is more salient tend to be more comfortable with ambiguity, diversity, and change, viewing these as opportunities for growth and self-development (Roccas et al., 2002).

As such, it is reasonable to expect that people with a weak growth-oriented value structure, and thus a greater need for self-protection, will perceive ethnic diversity as a threat to the cultural status quo and to their usual habits. This perception could facilitate the use of justification mechanisms for racially-based aggressive behavior at various levels, such as outgroup blaming and minimizing harm inflicted on individuals of other ethnicities. In contrast, individuals with a stronger growth-oriented mindset are hypothesized to have less recourse to moral disengagement: the greater relative importance placed on universalism and benevolence would encourage perspective-taking and empathic reasoning, assigning high importance to the well-being of others regardless of group differences (McFarland et al., 2012). Also, values centered around self-transcendence and a general focus on openness to others and diversity play crucial roles in discouraging prejudice (Souchon et al., 2017) and promoting intergroup contact with outgroups (Barni et al., 2020; Sagiv & Schwartz, 1995).

Consequently, this growth-oriented value mindset should undermine the basis for ethnic moral disengagement mechanisms and cognitive distortions grounded in ingroup–outgroup distinctions.

**H3.**  *Growth-oriented personal values are negatively linked with ethnic moral disengagement.*

## 2. Methods

### 2.1. Participants and Procedure

Participants were 413 undergraduate students from various first-year courses at a university in southern Italy, selected through a convenience sampling approach. The sample comprised 166 students who identified as male, 243 who identified as female, and 4 who did not disclose their gender. The mean age of participants was 19.60 years (SD = 1.46).

Participation was voluntary, and informed consent was obtained from all participants after they were provided with comprehensive information regarding the aims of the study. Prior to data collection, participants were explicitly reminded that their participation was not obligatory and that they could withdraw from the study at any time without any consequences. The experimental battery was structured through the Millisecond Inquisit precision testing platform, and data were collected via the Millisecond Inquisit Web cloud service for online administration of tests, with students responding from their home PCs. To ensure confidentiality, only the authors had access to the data, which were stored securely on the Millisecond EU Dublin-based repository in compliance with GDPR regulations. Upon completion of the data collection, all participants received a thorough debriefing that explained the objectives of the study, the methodologies employed, and the theoretical framework underpinning the research. All procedures were conducted in accordance with the ethical principles outlined in the Declaration of Helsinki and in full compliance with the ethical code of the AIP (Italian Psychology Association). The study protocol was reviewed and approved by the ethics committee of the university with which three of the authors are affiliated (Reference Code: ET-22-01).

### 2.2. Measures

#### 2.2.1. Ethnic Moral Disengagement

The ethnic moral disengagement (EMD) scale was used to assess the tendency to engage in moral disengagement mechanisms in the context of interethnic interactions (Lo Cricchio et al., 2022). Each item was rated on a 5-point Likert scale, with responses ranging from 1 (“Strongly disagree”) to 5 (“Strongly agree”). The results of the reliability analysis indicated that item 7 significantly detracted from the scale’s internal consistency. Furthermore, the item-total correlation for item 7 was comparatively low at 0.25, reinforcing the decision to exclude it from the scale. After its removal, the scale demonstrated robust internal consistency, evidenced by Cronbach’s alpha of 0.85 and McDonald’s omega of 0.89.

#### 2.2.2. Cognitive Reflection (CR)

The propensity of the individual to engage in slow and reflective reasoning when faced with a problem, rather than hastily providing the first answer that comes to mind, was evaluated through the four open-ended question items of the revised version of the Cognitive Reflection Test (Frederick, 2005), i.e., the Cognitive Reflection Test-2 (CRT-2) (Thomson & Oppenheimer, 2016). This specific version was selected for two reasons. First, it is less dependent on the individual’s mathematical abilities. Second, the original version of the CRT has become widely adopted, which could potentially influence the responses if the adolescent has encountered the items previously (Thomson & Oppenheimer, 2016). The four open-ended items were automatically coded with the help of the R package reflectR (version 2.1.3; Corbelli, 2024b). Each correct response was assigned one point, and the scores were then averaged, resulting in final test scores ranging from 0 to 1. A score of 0 indicated the absence of correct responses, while a score of 1 indicated that only correct reflective reasoning responses were given. The Cronbach’s alpha coefficient for the four test items was 0.66, while McDonald’s omega was 0.74.

#### 2.2.3. Growth-Oriented Personal Values

The 21 items of the PVQ-21 European Social Survey Value Scale (Schwartz, 2007; Schwartz et al., 2001) were used to assess the value salience of the respondent in relation to Schwartz’s theoretical framework. This instrument was chosen for its efficient administration and its cross-cultural validity (Schwartz et al., 2001). In particular, growth-oriented or self-expansive values were assessed through a higher-order value bipolar dimension, expressing anxiety-free motivations in contraposition to self-protective values. The statistical adjustment of raw scores, as recommended by the authors to correct for individual differences in response styles, and the computation of growth-oriented value scores (comprising hedonism, stimulation, self-direction, universalism, and benevolence) were carried out using the R package persval (version 1.1.1; Corbelli, 2024a). For the raw scores of growth-oriented values, Cronbach’s alpha was 0.83, and McDonald’s omega was 0.87.

#### 2.2.4. Inhibitory Control (SSRT; Stop Signal Reaction Time)

The ability to inhibit the prepotent response was assessed through the Stop Signal Task experimental procedure (Lappin & Eriksen, 1966; Logan & Cowan, 1984) for its effectiveness and robustness under various experimental conditions and delivered through the Inquisit Millisecond library on personal computers using audio headphones specifically selected to minimize external noise. The experimental procedure was administered in compliance with best practices outlined in the guidelines of Verbruggen and colleagues (Verbruggen et al., 2019) to evaluate the executive functioning of inhibitory capacity, providing a quantitative measure of the ability to inhibit an active behavioral response. In detail, subjects were asked to press a button to the left or right (either the E key or the I key on the keyboard) congruent with the direction of an arrow briefly presented on the screen and, upon presentation of a stop tone (25% of trials), participants are instructed not to respond to the appearance of the arrow in any way, inhibiting the automatic tendency to press the corresponding button. The acoustic signal was presented after a certain delay, varying this time frame adaptively during the test by following the level of the subject’s previous performance so that each participant was able to correctly inhibit his or her response in 50% of the total trials.

The main metric of the test is the inferred mean SSRT (Stop Signal Reaction Time), which is the mean time of the stop signal delay at which the subject was able to successfully inhibit the prepotent response in 50% of the cases. This assessment provides a quantitative measure of the ability to inhibit an already activated behavioral response, where longer SSRTs are indicative of worse inhibitory ability and a more impulsive response (Malesza & Ostaszewski, 2016; Shin et al., 2013). In the present study, this measure was reversed as a positive indicator of inhibitory control. The precision for estimating the time delay was achieved through the use of the experimental psychology measurement software Millisecond Inquisit Lab.

### 2.3. Planned Analyses

Initially, the raw data obtained from the Stop Signal Task were subjected to preprocessing protocols, as outlined in the guidelines established by Verbruggen and colleagues (Verbruggen et al., 2019). Descriptive statistics of the variables of interest were then calculated, followed by an examination of zero-order correlations.

Mardia’s test (Mardia, 1970) was then applied to assess the hypothesis of multivariate normality for the set of variables of interest, evaluating both skewness and kurtosis components. The presence of multivariate outliers was assessed by calculating Mahalanobis squared distances for each observation and comparing them with the critical value of the chi-square distribution at an alpha level of 0.001, with degrees of freedom equal to the number of variables.

Subsequently, the hypothesized theoretical relationships were empirically tested using a structural equation model (SEM), in which participants’ gender and age were included as covariates. All analyses were carried out in the R environment for statistical computing (R Core Team, 2023) using the following packages: haven (version 2.5.4; Wickham et al., 2015), psych (version 2.2.9; Revelle, 2022), mvnormalTest (version 1.0.0; Zhang et al., 2020), and lavaan (Rosseel, 2012).

## 3. Results

### 3.1. Preliminary Analyses

Descriptive statistics and zero-order correlations of the relevant variables are shown in Table 1. The results indicate that gender is positively associated with inhibitory control ability, with individuals who identify as female exhibiting better inhibitory control. Age did not correlate with any of the variables in the model. Mean ethnic moral disengagement was negatively associated with both the cognitive reflection measure and the relative importance attributed to growth-oriented values. In contrast, cognitive reflection was positively correlated with the importance placed on growth-oriented values. Finally, higher inhibitory executive functioning was associated with a decrease in moral disengagement and an increase in cognitive reflection.

Mardia’s test indicated a statistically significant departure from multivariate normality for both skewness (6387.11, *p* < 0.001) and kurtosis (115.63, *p* < 0.001). Moreover, 21 observations identified as multivariate outliers were excluded from the initial dataset, resulting in a final sample of 392 participants.

### 3.2. Structural Equation Model

To account for the non-normal multivariate distribution of observed variables, the structural equation model was tested using full information maximum likelihood estimation with robust (Huber–White) standard errors.

**Figure 1:**
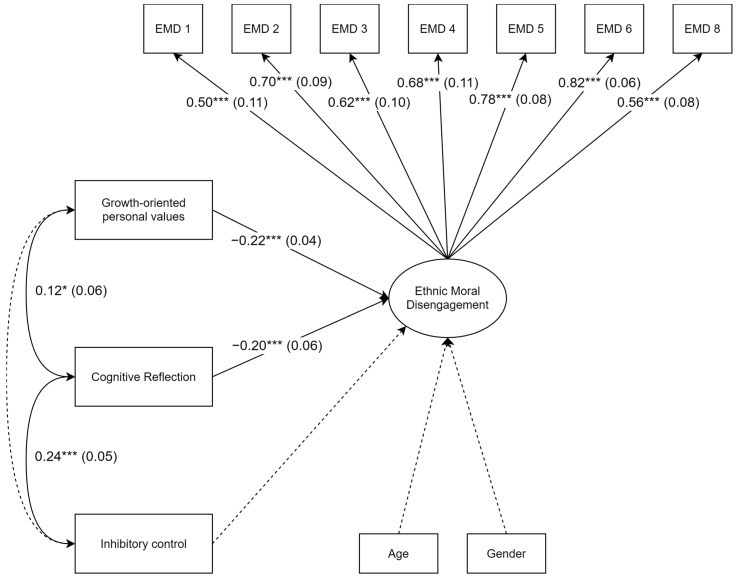
Structural equation model diagram with robust maximum-likelihood parameter estimates; standardized coefficients with their standard errors are displayed. EMD 1 to EMD 8 represent the respective items of the ethnic moral disengagement (EMD) scale. Solid lines represent significant paths (*p* < 0.05) and dotted lines represent non-significant paths. * *p* < 0.05, *** *p* < 0.001.

The plausibility of the hypothesized relationships was evaluated using several model fit indices, which confirmed that the model fits the empirical data well according to the cutoff values proposed by Kline (Kline, 2016): χ^2^ = 51.66, df = 32, *p* = 0.015; CFI = 0.97; TLI = 0.96; RMSEA = 0.05, 90% CI = [0.000, 0.082], *p* = 0.480; SRMR = 0.04. Figure 1 displays the standardized coefficients and standard errors.

Contrary to what was hypothesized (H1), the measure of inhibitory control was not found to be associated with the level of ethnic moral disengagement. Instead, the results show that an increase in cognitive reflection is significantly associated with a decrease in ethnic moral disengagement (H2; β = −0.20, SE = 0.06, *p* = 0.001) and that a higher relative importance placed on growth-oriented personal values is linked to a lower level of moral disengagement (H3; β = −0.22, SE = 0.04, *p* < 0.001). However, it was correlated with cognitive reflection: those with better inhibitory control also had a greater tendency to engage in reflective reasoning (r = 0.24, SE = 0.05, *p* < 0.001). In contrast, the score obtained in growth-oriented values did not correlate with inhibitory control but did correlate with cognitive reflection, suggesting that more reflective individuals also attach greater importance to these values (r = 0.12, SE = 0.06, *p* = 0.044). In total, the model explained 12% of the variability in ethnic moral disengagement (R^2^ = 0.12).

## 4. Discussion

Ethnic moral disengagement is a specific type of moral disengagement recently theorized (Lo Cricchio et al., 2022), representing an emerging research area in the literature on moral functioning that underlies social behavior toward outgroup members. This paper contributes to the literature by focusing on the antecedents of ethnic moral disengagement that, at the individual level, can help explain the nature of this construct and, in this way, also point to possible ways of action to intervene on such a detrimental cognitive distortion against immigrants. Specifically, the present study accomplished this by associating the explanatory power of three completely different individual dimensions of analysis, both in purpose and in assessment methodology. Consideration was given to (1) the results of a task-based objective experimental procedure to assess inhibitory control with the necessary methodological rigor required by such a laboratory assessment (Lappin & Eriksen, 1966; Logan & Cowan, 1984; Verbruggen et al., 2019); (2) the results of a cognitive test with an open-ended question to assess cognitive processing style with only one possible correct answer, coded through an automated procedure (Corbelli, 2024b; Thomson & Oppenheimer, 2016); and (3) the results of a self-report assessment to measure personal values via a cross-culturally validated instrument (Schwartz, 2007; Schwartz et al., 2001), appropriately processed as required by the relevant literature. This diversity of assessment approaches was necessary to capture, as far as possible, the inherent complexity of the different dimensions underlying ethnic moral disengagement, aiming to identify its individual behavioral, cognitive, and motivational components. Overall, from a broader perspective, the results indicate a negative relationship between the presence of a growth-oriented mindset and ethnic moral disengagement. Such a growth mindset would seem to arise from the association between slow and careful processing of reality, on the one hand, and the direction of the self-system toward a perspective of growth and discovery of diversity when confronted with uncertainty on the other. It is important to recognize how these two dimensions that identify such a mindset are malleable and learnable. The findings suggest a dispositional element in the tendency toward reflective reasoning, as indicated by its positive link with inhibitory control. Nevertheless, while basic inhibitory ability by its nature is relatively stable in adulthood (Kuntsi et al., 2006; Shin et al., 2013), cognitive reflection can be exercised and learned, thus incentivizing and enhancing a slower and less reactive reflective ability when faced with social information related to the ethnic characteristics of individuals.

More in-depth, the hypothesized direct association between inhibitory control and ethnic moral disengagement was not supported by empirical evidence for the sample considered. Although its essential role in the regulation of impulsive behavior is well known in the neuropsychological literature, and although the general literature on MD seems to suggest such a connection (Gülseven et al., 2023; Paciello et al., 2023), inhibitory control may not play a significant role in this case toward ethnic moral disengagement since moral disengagement is not an acted-out behavior per se, but it is a set of dynamic cognitive processes that temporarily suspend moral self-regulation and self-sanction. As the prepotent response inhibition measure is an assessment of an executive function that is fundamentally motor/behavioral in nature, it may not have a direct impact on this set of cognitive mechanisms. Moreover, such inhibitory capacity, although crucial and fundamental in daily life, certainly allows individuals to pause before acting automatically, but it does not specify what courses of action individuals will take. The cognitive space created by physiological inhibitory control can indeed be utilized for reflective, slow, and attentive cognitive processing; however, it is not guaranteed to be used in this way. While a certain level of inhibitory control is necessary for a reflective cognitive mode, it is not sufficient by itself. Theoretically, this space could also be occupied by rapid, simplified responses that rely on automatic cognitive shortcuts.

Indeed, from the present study, a crucial dimension appears to be cognitive reflection. The negative association between reflective reasoning and ethnic moral disengagement highlights the importance of a cognitive style that involves systematic and effortful information processing rather than relying on the first plausible cognitive response or seeking quick solutions to reduce uncertainty and ambiguity. Individuals inclined toward reflective reasoning are less likely to endorse indicators of moral disengagement related to aggression toward other ethnic groups. These reflexive cognitive strategies could be a key to metacognitively examining automatic biases and challenging simplified and stereotyped interpretations that justify harmful behaviors. Cognitive reflection can help overcome low-effort first impressions arising from heuristic thought processes, which often result in stereotyping by associating outgroups with negative characteristics in a static and uncritical way, thereby simplifying complexity and diminishing moral reflection on us/them categories (Blanchar & Sparkman, 2020). Such propensity for reflective reasoning, then, could be a necessary component for self-monitoring and self-reflection, whose importance Bandura recognizes in moral self-regulation (Bandura et al., 1996). Under conditions where this slow and careful cognitive style is less prevalent, moral disengagement mechanisms may manifest more strongly due to difficulties in self-monitoring and self-observation, preliminary to moral control.

In addition, the findings on the motivational dimension related to growth-oriented values provide a significant addition to the explanation of the antecedents to moral disengagement. Individuals who emphasize an association of benevolence, universalism, self-direction, and stimulation as guiding values tend to be less prone to ethnic moral disengagement. Interestingly, beyond self-transcendence values, whose protective role against moral disengagement is recognized (Albouza et al., 2020), growth-oriented values also encompass self-direction and stimulation. This suggests that such a value orientation not only views others as worthy of moral consideration but also includes a self-related component of openness and personal growth through interaction with what is new and different. Together, these growth-oriented values provide a moral motivational backbone, but they also have a direct and positive relationship with cognitive reflection: individuals who prioritize values further from this growth-oriented mindset may be more inclined to perceive outgroup members as threatening or deviant, leading to the use of quick cognitive distortions that simplify responsibility for harm on an ethnic basis and justify discriminatory behavior.

Overall, reflective reasoning facilitates a richer metacognitive inspection of thought processes, while growth-oriented values provide moral guidelines in the face of anxiety and uncertainty arising from diversity: both components can lead individuals to avoid resorting to a kind of cognitive disengagement that exploits intergroup differences. With both of these components, individuals have the opportunity to more effectively expose and then dismantle the cognitive distortions supporting moral disengagement.

From an intervention perspective, combining the cultivation of reflexive cognitive strategies (e.g., engaging in structured debates to foster critical thinking, using case studies for training in the reflective evaluation of social information, and participating in role-playing exercises to practice taking multiple perspectives) with efforts to elevate growth-oriented values (e.g., educational programs to recognize that novelty perceived as different can be an opportunity and workshops that cultivate appreciation for diverse perspectives (D’Errico et al., 2024)) could strengthen their effects in countering moral disengagement. This two-way approach, one reflective–cognitive and another motivational–moral, could thus contribute to the development of such a growth mindset. Additionally, integrating training in impulse control can further enhance this strategy by providing a foundation for slow cognitive processing, which helps counteract heuristic thinking.

Future studies could extend this line of research by examining whether and how these three factors, i.e., inhibitory behavioral control, reflective cognitive style, and growth-oriented values, are related to other forms of moral disengagement besides the ethnic one. It would be interesting to determine whether the pattern observed here generalizes to moral disengagement in domains such as online environments, where moral disengagement may be facilitated by anonymity, depersonalization (Paciello et al., 2020), stereotypes (Bosco et al., 2023), polarization, and conflict (Poggi et al., 2011; D’Errico & Poggi, 2014; Kubin & Von Sikorski, 2021). Another interesting direction suggested by this study involves the use of longitudinal approaches to investigate how changes in reflective reasoning skills or changes in value priorities temporally influence trajectories of moral disengagement, adopting a research design that would help clarify causal directions and potential synergy among these factors. In future studies, it might also be useful to consider how contextual factors, such as group norms and the cultural characteristics of the social environment, contribute to explaining ethnic moral disengagement. Understanding how these factors interact with cognitive and motivational variables could enhance the model’s explanatory power regarding this construct.

While it makes a novel contribution to understanding the mechanisms of ethnic-based moral disengagement, the present study has some limitations. First, the data on which this study is based are purely correlational. Although a structural equation modeling approach was used to test the hypothesized relationships, causal inferences cannot be drawn conclusively. As indicated, longitudinal and/or experimental designs would be needed to recognize a causal effect. Moreover, since the sample consists of university students from a specific Italian region collected through convenience sampling, it may not fully represent larger populations. Therefore, replications and cross-cultural comparisons are essential to assess the generalizability of these findings.

## Figures and Tables

**Table 1:** Descriptive statistics and zero-order correlation for the relevant variables.

	M	SD	1	2	3	4	5
1. Gender	-	-	-				
2. Age	19.60	1.46	−0.13 **	-			
3. EMD	1.20	0.45	−0.04	−0.01	-		
4. CR	0.35	0.32	0.00	0.00	−0.28 ***	-	
5. VAL	0.40	0.36	0.08	−0.05	−0.28 ***	0.11 *	-
6. INHIB	290.73	127.83	0.15 **	0.04	−0.14 *	0.24 ***	0.05

EMD: ethnic moral disengagement; CR: cognitive reflection; VAL: growth-oriented values; INHIB: inhibitory control. * *p* < 0.05, ** *p* < 0.01, *** *p* < 0.001.

## Data Availability

The dataset supporting the results of this study is available upon motivated request to the corresponding author.

## Abbreviations

The following abbreviations are used in this manuscript:

MD	moral disengagement
EMD	ethnic moral disengagement
